# Rethinking Serotonin in Depression: Toward a Modern Clinical Framework

**DOI:** 10.7759/cureus.111172

**Published:** 2026-06-19

**Authors:** Alexander Sheppert

**Affiliations:** 1 Internal Medicine, Legacy Health, Vancouver, USA

**Keywords:** antidepressants, depression, evidence-based medicine, primary care, selective serotonin reuptake inhibitors, serotonin

## Abstract

Research has failed to demonstrate that depression is caused by a deficit in central serotonin activity. Umbrella reviews show no consistent association between serotonin markers and major depressive disorder (MDD), and routine clinical practice does not measure serotonin before treatment. Selective serotonin reuptake inhibitors (SSRIs) produce statistically significant but modest improvements over placebo in pooled analyses that include multiple antidepressant classes, with benefit concentrated in patients with severe depression and diminished in mild-to-moderate cases. SSRIs do provide meaningful relief for some patients and remain an important treatment option, particularly when access to psychological therapies is limited. Emerging mechanistic models suggest that SSRIs act less by correcting a chemical imbalance and more by reducing negative affective bias and modestly enhancing neuroplasticity, creating a window in which psychosocial and behavioral interventions can take hold. Because SSRIs are linked to common adverse effects, including sexual dysfunction, weight gain, and sleep disturbance, their routine use as a universal first-line treatment warrants re-examination. Recent international guidelines from the World Health Organization (WHO), the National Institute for Health and Care Excellence (NICE), and the American Psychiatric Association (APA) now prioritize psychological therapies, structured exercise, and combination approaches over antidepressant monotherapy for non-severe depression. This editorial proposes a pragmatic, patient-centered framework for general internists that accounts for real-world access barriers, screens for reversible contributors, offers evidence-based non-pharmacological options where feasible, and repositions SSRIs as adjuncts or facilitators rather than default interventions.

## Editorial

A 22-year-old patient asks whether a “chemical imbalance” explains her depression. The story is familiar: depression means “low serotonin,” and a daily tablet restores it. This narrative has been propagated in clinical training and patient education because it offers a simple, biomedical explanation for suffering. Yet, it is not what the evidence shows.

If depression were truly a serotonin deficiency disorder, clinicians would measure serotonin (or a reliable downstream marker) before prescribing. They do not, because available assays correlate poorly with depressive symptoms. A 2022 umbrella review in Molecular Psychiatry concluded that the main areas of serotonin research provide no consistent evidence linking lowered serotonin to depression [1]. Cerebrospinal fluid studies similarly found no significant abnormalities in serotonin metabolites in major depressive disorder (MDD) [2]. Thus, the core premise of the “chemical imbalance” model is unsupported.

At the same time, a potentially harmful paradox persists in practice: we tell patients their serotonin is too low, we do not measure it, we raise it pharmacologically, and sometimes, we raise it far enough to cause serotonin toxicity, all without reliably treating their depression [3]. The serotonin-deficit narrative should be retired and replaced with a framework that better fits contemporary evidence and clinical guidelines. This editorial examines the evidence underlying selective serotonin reuptake inhibitor (SSRI) use, the emerging mechanistic models that explain their effects, and the growing guideline consensus favoring a more flexible, patient-centered approach to depression treatment in primary care.

Collapse of the serotonin-deficit narrative

The first challenge to the deficit model is temporal. SSRIs increase synaptic serotonin within hours, but mood improvement, when it occurs, usually appears only after four to six weeks. If depression were caused by low serotonin, the clinical response should mirror the pharmacokinetics. The lag implies that intermediate processes, such as emotional learning or neuroplastic change, mediate benefit.

A second challenge is phenomenological: the same drug class is used for MDD and for several anxiety disorders. A unitary serotonin deficit does not convincingly explain both anxious hyperarousal and depressive psychomotor slowing.

Finally, toxicity undermines the model. Patients can be titrated to cumulative serotonergic loads that risk serotonin syndrome while still being depressed [3]. A theory that cannot distinguish “not enough serotonin” from “too much serotonin” is clinically weak.

Clinical data on antidepressants and SSRIs

It is important to note at the outset that several landmark meta-analyses in this area pooled data across multiple antidepressant classes, not SSRIs alone, and readers should interpret the findings accordingly. While SSRIs are the focus of this editorial, the broader antidepressant literature provides essential context.

The 2018 network meta-analysis by Cipriani et al. in The Lancet examined 21 antidepressant drugs (including SSRIs, serotonin-norepinephrine reuptake inhibitors (SNRIs), and tricyclic antidepressants (TCAs)) and found that approximately four in 10 patients met response criteria versus about three in 10 on placebo, yielding an absolute difference of roughly 10% [4]. On rating scales, this typically translates to a two-to-three-point mean difference, which is statistically significant but may be below the threshold of clinical salience for many patients.

The 2010 patient-level meta-analysis by Fournier et al. in JAMA similarly examined multiple antidepressant medications and showed that the drug-placebo difference becomes substantial only in very severe depression; in mild-to-moderate cases, the difference was minimal or non-existent [5]. This suggests that the modest average effect in large datasets is largely driven by a sicker subgroup.

Methodology further narrows the signal. Trials often use inert placebos. Because SSRIs cause noticeable side effects, many participants can guess their allocation, which inflates apparent efficacy. When active placebos that mimic side effects are used, the drug-placebo gap shrinks substantially [6]. Selective publication of positive trials adds another layer of inflation [7]. Kirsch and colleagues have argued that the larger effect in severe depression largely reflects reduced placebo responsiveness in those patients, not a stronger drug effect [8].

That said, SSRIs should not be dismissed. They have a well-established safety and tolerability profile relative to older antidepressant classes such as TCAs and monoamine oxidase inhibitors (MAOIs), which is one reason they became first-line pharmacotherapy. Individual patients do experience clinically meaningful improvement, and for patients with severe depression or those who cannot access psychotherapy, SSRIs remain an important and sometimes essential treatment option [4,9].

Antidepressants, including SSRIs, work modestly on average, for some patients, in carefully selected trial populations, and especially in the most severe cases. That is a legitimate, but limited, clinical role that should be weighed against alternatives.

Emerging mechanistic frameworks

Because the serotonin-deficit model does not fit the data, attention has shifted to what SSRIs enable rather than what they “replace.”

Affective-Bias Model

Before patients report feeling better, SSRIs can dampen amygdala reactivity to negative stimuli and shift attention away from negative emotional cues [10]. This creates a window in which patients can relearn less depressive interpretations of their environment, but the relearning itself takes weeks, which explains the clinical lag.

Neuroplasticity Model

Depression is associated with neuronal atrophy and reduced synaptic connectivity. Agents that rapidly increase plasticity, such as ketamine and psilocybin, acutely upregulate brain-derived neurotrophic factor (BDNF) and promote synaptogenesis, producing rapid antidepressant effects, plausibly by reopening periods of environmental learning [11]. Conventional SSRIs raise BDNF as well, albeit more modestly and more slowly [12]. Under this view, the SSRI does not fix a serotonin shortage; it makes neuronal pathways more malleable, loosening rigid depressive networks so that psychotherapy, social contact, exercise, or light therapy can take hold. This modern framing supports a step that clinicians already take implicitly: using an SSRI as a bridge to more definitive interventions.

Adverse effects of SSRIs

SSRIs are not benign. Reported rates for common effects are higher than many patients are told (Table 1). Sexual dysfunction is reported in over half of users (56%-75%) and can persist after discontinuation [13,14]. Clinically meaningful weight gain (greater than 5% body weight) occurs in 55%-65% with longer use [15]. Somnolence, sweating, sleep disruption, cognitive symptoms, hyponatremia, and increased bleeding risk are all well described [13,16-18]. Serotonin syndrome, though uncommon, is potentially fatal and proves that “more serotonin” is not unconditionally therapeutic [3]. Given that the mean drug-placebo difference is modest, the risk-benefit balance will be unfavorable for many patients, especially those with mild illness.

**Table 1:** Common adverse effects of SSRIs SSRI: selective serotonin reuptake inhibitor; OR: odds ratio

Adverse effect	Incidence rate	Notes	Source
Sexual dysfunction	56%-75%	Includes decreased libido, anorgasmia, and erectile dysfunction; may persist after discontinuation	[13,14]
Weight gain (>5% body weight)	55%-65%	With longer-term use	[15]
Somnolence	Up to 59%	Direct sedative effects	[13]
Excessive sweating	29%	Autonomic dysregulation	[13]
Sleep disruption/insomnia	17%-28%	Alters sleep architecture	[16]
Cognitive symptoms	>20%	Difficulty concentrating, mental slowing	[13]
Nausea	10%-26%	Increased serotonin in gut	[13]
Hyponatremia	6% (0.82% clinically significant)	Syndrome of inappropriate antidiuretic hormone secretion (SIADH) mechanism	[17]
Increased bleeding risk	OR 1.7%-2.0	Impaired platelet aggregation	[18]
Serotonin syndrome	Uncommon	Potentially fatal; risk increases with polypharmacy	[3]

Alternative and adjunctive treatments

Non-pharmacological options are not weaker than SSRIs; in several high-quality syntheses, they are comparable or stronger. A 2024 network meta-analysis by Noetel et al. in BMJ examined 218 randomized controlled trials (RCTs) comprising 14,170 participants and reported that jogging, yoga, and cognitive-behavioral therapy (CBT) produced larger effect sizes than SSRIs, and that SSRIs were close to placebo for non-severe depression [19]. An umbrella review by Mavranezouli et al. in EClinicalMedicine the same year synthesized 11 meta-analyses covering psychological and behavioral interventions and reached a similar conclusion: these interventions tended to outperform medication in mild-to-moderate cases, and combined approaches were best overall [20]. The 2022 network meta-analysis by Recchia et al. in the British Journal of Sports Medicine analyzed 21 RCTs and found exercise to be at least as effective as antidepressants for non-severe depression [21].

Lifestyle and somatic measures add further leverage: nutrition-informed care [22], sleep-improvement interventions with medium-to-large effects on depressive symptoms across 65 RCTs [23], and light therapy with moderate benefit across 25 RCTs [24].

However, the efficacy of these interventions must be weighed against real-world implementation challenges. Behavioral change programs have high attrition rates. Structured psychotherapy requires access to trained therapists, which is limited by cost, geographic availability, and long wait times. These barriers disproportionately affect underserved populations. Even when patients are referred to mental health specialists, many do not follow through, and those who do rarely complete what would be considered a therapeutic “dose” of psychotherapy [25]. Exercise prescriptions, while effective in trials, depend on motivation and physical capacity that depression itself undermines. A patient who cannot get out of bed will also not jog or attend CBT.

For clinicians in resource-limited primary care settings, these barriers are not theoretical; they are the daily reality of practice. A prescription for a generic SSRI is something a primary care physician can offer immediately, and antidepressant adherence is often higher than adherence to psychotherapy referrals, even among patients who express a preference for therapy [26]. This pragmatic reality must be part of any honest treatment framework.

Here, a short-term SSRI reframed as a facilitator of engagement, rather than a biochemical correction, makes clinical sense: it lowers the activation barrier enough for the patient to participate in the behavioral and psychosocial interventions that drive sustained recovery.

Current clinical practice guidelines

Recent guidelines now reflect the evidence shift toward multimodal treatment: The WHO 2023 guidelines recommend structured psychological interventions as first-line treatment and note that antidepressants should be used when such interventions are unavailable; the overall certainty of evidence for SSRIs is rated very low [27]. The NICE 2022 guidelines (United Kingdom) state: “Do not routinely offer antidepressants as first-line treatment, unless that is the person’s preference.” They recommend offering a menu of options using shared decision-making [28]. This is a marked change from the 2009 guidance, which was more medication-forward. The APA 2010 guidelines (United States) identify both psychotherapy and a second-generation antidepressant as acceptable first-line choices, with combination approaches when a faster or fuller response is needed [29].

Notably, no major clinical guideline currently recommends SSRIs as the sole or automatic first-line treatment for all severities of depression. A modern clinical framework should therefore adopt the same starting point: a flexible, individualized approach.

A proposed framework for depression management

**Figure 1:**
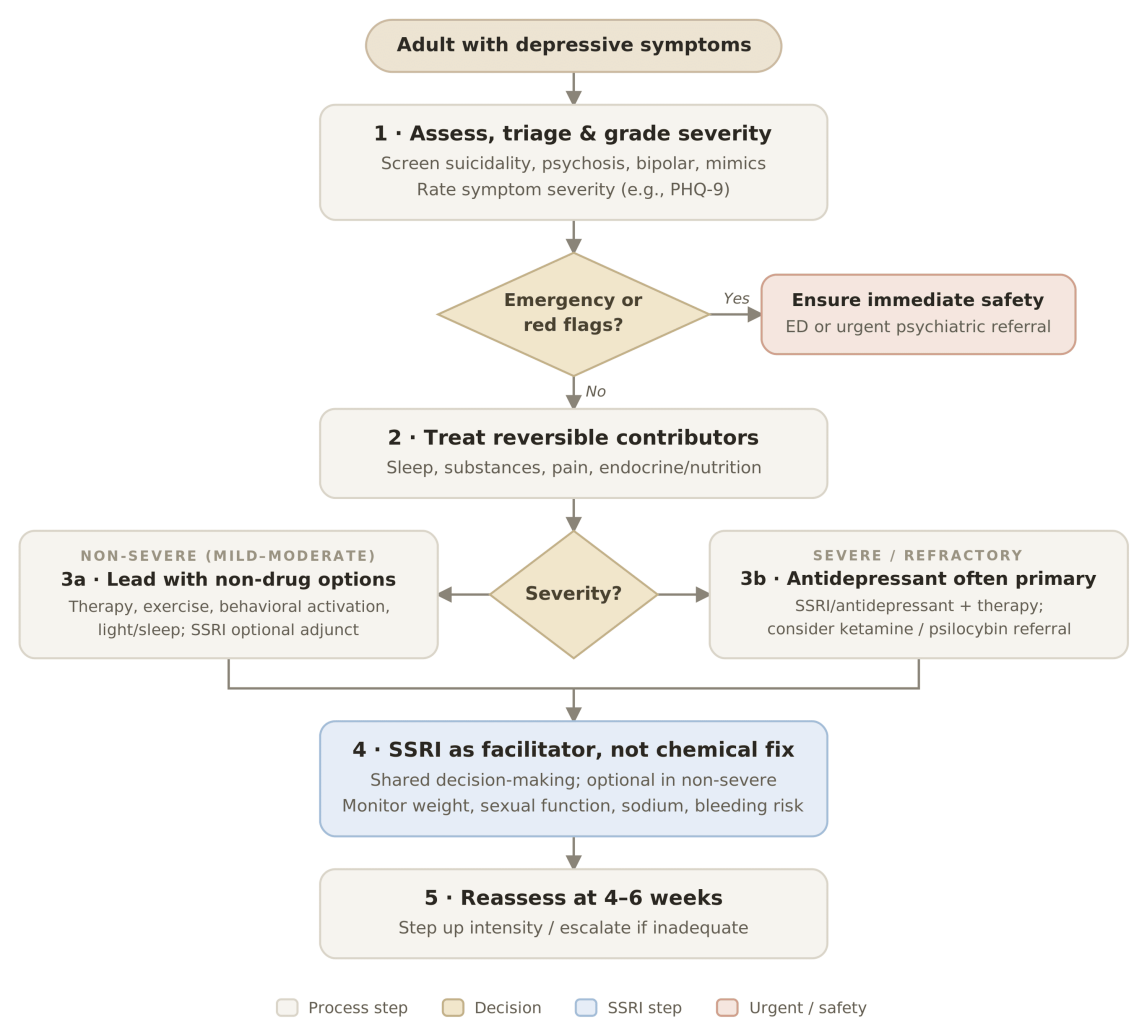
A proposed framework for depression management in primary care A severity-matched decision tree operationalizing the framework described in the text. After assessment and severity grading, and treatment of reversible contributors, management is matched to severity: non-pharmacological options lead for non-severe (mild-to-moderate) illness (3a), whereas an antidepressant is often primary for severe or refractory illness (3b). An SSRI, when used, is positioned as a facilitator rather than a chemical correction, and response is reassessed at 4-6 weeks. Shading denotes node type (see key). ED: emergency department; PHQ-9: Patient Health Questionnaire-9; SSRI: selective serotonin reuptake inhibitor

The following framework is intended not as a novel paradigm but as a pragmatic synthesis of current guideline recommendations, adapted for the realities of general practice (Figure 1). It recognizes that the ideal and the feasible often diverge, particularly in primary care settings with limited mental health resources.

Initial Assessment and Triage

Screen for suicidality, psychosis, bipolarity, and medical mimics, and grade symptom severity (e.g., Patient Health Questionnaire-9 (PHQ-9)). Address emergencies first.

Investigate and Treat Reversible Contributors

Sleep disturbance, acute psychosocial stressors, alcohol or substance use, pain, and endocrine or nutritional factors (omega-3 intake, vitamin D, and anemia) should be actively corrected.

Match Treatment to Severity, Scaled to Access

For non-severe (mild-to-moderate) depression, lead with non-pharmacological options: where available, these include CBT or other structured psychotherapy, prescribed physical activity (start low, progress), behavioral activation and social reconnection, and light/sleep interventions, with an SSRI as an optional adjunct. Where these are not accessible because of cost, geography, wait times, or patient capacity, clinicians should still discuss them as goals and help patients access lower-barrier alternatives such as digital CBT platforms, community exercise programs, or guided self-help resources. For severe or refractory depression, an antidepressant is often appropriate as a primary intervention, combined with psychotherapy and, where available and appropriate, referral for neuroplasticity-promoting interventions (ketamine; psilocybin-assisted protocols) grounded in the plasticity model.

Reposition SSRIs as Facilitators, Not Defaults

Present SSRIs as optional adjuncts with modest average benefit, substantial heterogeneity of response, and a defined side-effect burden. Use shared decision-making. In settings where non-pharmacological options are truly unavailable, an SSRI may reasonably serve as a first step, but the framing should be that the medication is opening a window for recovery, not correcting a chemical deficiency. Track weight, sexual function, sodium (in higher-risk patients), and bleeding risk.

Reassess and Escalate

Reassess response at 4-6 weeks and step up intensity or escalate care for an inadequate or refractory response.

Limitations

This editorial draws heavily on meta-analyses and umbrella reviews, which themselves contain heterogeneous trials, short follow-up windows, industry sponsorship, and variable definitions of depression. Several key meta-analyses cited here pooled multiple antidepressant classes; SSRI-specific effect sizes may differ modestly from pooled estimates. Long-term comparative data on medication-versus-non-medication strategies remain sparse. The access barriers to non-pharmacological treatment discussed here vary substantially by healthcare system and geography. Nonetheless, both the weight of current evidence and the direction of current guidelines support de-emphasizing the serotonin-deficit narrative and adopting a more flexible approach.

Concluding remarks

The serotonin-deficit narrative offered a convenient story but not an accurate one. Primary care physicians prescribe the majority of antidepressants in the United States; we should therefore lead the shift toward guideline-concordant care that prioritizes psychological and behavioral interventions where accessible, while using SSRIs judiciously as bridges to recovery rather than as biochemical corrections. For patients in settings where therapy and lifestyle interventions are genuinely out of reach, SSRIs remain a reasonable option. Even so, the explanation we give patients and the expectations we set should match the science, not the marketing. Our patients deserve explanations that reflect the evidence and treatment plans that account for both the evidence and their circumstances.
